# History of psychiatry in Nepal

**DOI:** 10.1192/bji.2021.51

**Published:** 2022-02

**Authors:** Rakesh Singh, Anoop Krishna Gupta, Babita Singh, Pragyan Basnet, S. M. Yasir Arafat

**Affiliations:** 1 Independent Mental Health Researcher, and Visiting Faculty Member, Department of Public Health, KIST Medical College, Tribhuvan University, Kathmandu, Nepal. Email: rakes4r@gmail.com; 2 Lecturer, Department of Psychiatry, National Medical College, Birgunj, Nepal; 3 Professor and Vice-Principal, Department of Psychiatric Nursing, National Medical College, Birgunj, Nepal; 4 Medical Student, School of Medicine, Patan Academy of Health Sciences, Lalitpur, Nepal; 5 Assistant Professor, Department of Psychiatry, Enam Medical College and Hospital, Dhaka, Bangladesh

**Keywords:** History of psychiatry, low- and middle income countries, landmarks of psychiatric services, Nepal, ups and downs of mental healthcare

## Abstract

The history of psychiatry as a discipline in Nepal has been poorly studied. We have attempted to summarise historical landmarks to explore how it began and its evolution over time in relation to contemporary political events. Although Nepal has achieved several milestones, from establishing a psychiatric out-patient department with one psychiatrist in 1961 to having more than 500 psychiatric in-patient beds with 200 psychiatrists by 2020, the pace, commitment and dedication seem to be slower than necessary: the current national mental health policy dates back to 1996 and has not been updated since; there is no Mental Health Act; the number of psychiatric nurses and in-patient psychiatric beds has increased only slowly; and there is a dearth of professional supervision in rehabilitation centres. Thus, despite making significant progress, much more is required, at greater intensity and speed, and with wide collaboration and political commitment in order to improve the mental health of all Nepali citizens, including those living in rural areas and or in deprived conditions.

## Background

Nepal is one of the developing nations in South Asia, bordering two rapidly growing economies, China to the north and India to the east, west and south. The country became an independent nation in 1923. According to the 2020 United Nations Human Development Report, Nepal ranks 142nd out of 189 countries on the Human Development Index, and the 2021 World Happiness Index places the country in 87th position out of 149 countries.^1,2^ Nepal's population is 29.14 million, as of mid-2020, according to UN data. Most (78.6%) people live in villages and rural areas which are often deprived of specialist healthcare, including mental health services. In 2016, an estimated 30% of the Nepalese population suffered from psychiatric problems, but over 90% did not have access to mental health services.^3^ The prevalence of poor mental health is rising, accelerated by the coronavirus disease (COVID-19) pandemic, which has disrupted mental health services, reduced use of mental healthcare and caused economic hardship. A novel three-tier (central, federal/provincial and local) healthcare delivery system has recently been adopted nationwide, but it has limited resources and infrastructure. Mental health remains underfunded; it receives less than 1%^3^ of Nepal's total healthcare budget and is supported by only 2% of medical and nursing training. Nationally there are around 500 beds for people with mental disorders (i.e. 1.5 beds per 100 000 people), just 200 psychiatrists (0.68 psychiatrist per 100 000 people) and 50 psychiatric nurses (0.17 psychiatric nurse per 100 000 people).^4^ Moreover, most of the available mental health services are concentrated in urban areas, where only 21.4% of the Nepalese population resides.^3^

## Ancient practices (before 1961: the monarchial era)

Historically, mental asylums were often the first point of contact for those seeking mental healthcare in most nations, but until relatively recently patients in Nepal needed to go to towns in the northern part of India (including Ranchi, Gorakhpur and Lucknow) to seek mental treatment. Many people used alternative medicines, including Ayurveda and Bhutvidhya, for the management of mental disorders. Bhutvidhya, popularly known as Ayurvedic psychology, has been in existence in Nepal for centuries and uses counselling as a means of promoting mental well-being.^5^

## The era of modern psychiatry

The first psychiatric service in Nepal was founded after the December 1960 coup d’état by King Mahendra.

A psychiatric out-patient department (OPD) was established in 1961 at Bir Hospital, when Nepal's first psychiatrist returned after completing his professional training in Great Britain. The Bir general hospital was established in 1889 by the then Prime Minister Bir Shamsher Jung Bahadur Rana and consisted of a mere seven beds and five staff. The psychiatric OPD was extended by a psychiatric in-patient unit in 1965, which comprised five beds, and further increased by twelve beds in 1971.^6^ In 1976, a neuropsychiatric unit with ten beds was created for military personnel and their families at the Tri-Chandra Military Hospital. In the same year, a rehabilitation centre for drug users in Nepal was established by the Reverend Father Thomas Edward Gafney, a Jesuit priest (later the victim of a notorious murder).

Subsequently, non-government organisations (NGOs) supported the development of mental rehabilitation services. A psychiatric OPD and a 12-bed in-patient psychiatric service were created in Tribhuvan University Teaching Hospital in Kathmandu in 1986 and 1987 respectively. In 1984, the OPD at Bir Hospital was incorporated into a new mental hospital with 25 in-patient beds at Lagankhel, Lalitpur, the first and only central-level mental hospital in Nepal, popularly known as Lagankhel Mental Hospital.^6^ In 2003, the hospital extended its total beds to 50; there has been no further expansion. On average, 175 patients visit the OPD daily, many of them requiring admission. In 1983–1984 some NGOs also started to work in the sector of intellectual disability; however, much more needs be done in this field.

## Community mental healthcare

The history of community mental health services dates to 1983. That is when healthcare workers in and around Bhaktapur were trained in mental health, leading to the establishment of a satellite mental health clinic for the cases referred by them. The United Mission to Nepal started its mental health programme in 1984 as a 5-year pilot project in Lalitpur, training paramedics to provide a primary level of care. This proved to be both affordable and feasible as a means of providing services in a resource-deprived state. After its success, a similar mental health project was launched by the Tribhuvan University Teaching Hospital in 1989 with the support of international NGOs. Community mental health programmes were replicated in Morang, Kaski and Banke; later, the Kaski programme was expanded to include Syangja District. In 2017, funding for a community mental healthcare package extended these services to remote areas and nowadays an NGO continues this work in 26 districts.

Some academic courses in psychiatry were established during the 1996–2006 Maoist insurgency. The qualification Doctor of Medicine (MD) in Psychiatry was initiated at the Institute of Medicine (IOM) in 1997 and at the BP Koirala Institute of Health Sciences (BPKIHS) in 1999. In 1998, the IOM created a 2-year residency programme in clinical psychology and in 2000 it established a Bachelors programme in Psychiatric Nursing at the Maharajgunj Nursing Campus. The Norwegian organisation Redd Barna (Save the Children, Norway) provided mental health training to teachers and nurses. BPKIHS established a Master of Nursing programme in psychiatry in 2008; some students travel to the National Institute of Mental Health and Neurosciences, Bengaluru, India, which provides a 1-year diploma in nursing. Currently, the MD in Psychiatry programme runs in 12 out of 20 Nepalese medical colleges. A Masters programme in Psychiatric Nursing exists at four universities (Tribhuvan University, Kathmandu University, BPKIHS and the Patan Academy of Health Sciences). Although there are child and adolescent clinics at Kanti Children's Hospital in Kathmandu, there is no Doctor of Medicine (DM) programme in this psychiatric discipline.^7^

The Narcotic Drugs Control Act was framed in 1976 and amended in 1981 and 1987. The Drug Abuse and Demand Reduction Project was initiated in 1995 and several demand-reduction activities are carried out by national and international NGOs. Prevailing Nepalese legislation has not decriminalised the acquisition, use or possession of illegal drugs for personal use.^8^ In response to the problem of drug misuse, several rehabilitation centres have been set up by NGOs. The number of hard drug users increased from 46 309 to 130 424 between 2013 and 2019, resulting in pressure on limited resources provided by rehabilitation centres.^9^ Currently, these centres are unable to provide adequate specialist treatment owing to a lack of psychiatrists. Most of them are supervised by medical officers.

## National Mental Health Strategy and Action Plan 2020

In 1984, an epidemiological field survey in the Kathmandu valley reported the prevalence of mental illness to be 14%. Subsequently, it has been estimated that 18% of the non-communicable disease burden is due to mental illness.^10^ Surveys into specific issues such as the prevalence of substance use disorders and mental disorders in children have not been planned at a national level, mainly because of political factors, but recently a general national mental health survey has been initiated by the Nepal Health Research Council with governmental support.^11,12^ It has so far extended to three of the seven provinces of Nepal. The Ministry of Health and Population has endorsed a new National Mental Health Strategy and Action Plan (November 2020)^4,13^ to combat increasing mental health problems, including those arising from the current COVID-19 pandemic. Its objectives are to provide free, approachable and basic mental health services through multi-sectoral collaboration. It also emphasises the need for research into evidence-based practice and the dissemination of information to combat supernatural beliefs and myths about mental illness.

Nepal does not have a separate Mental Health Act.

## Recent advances

With the advent of digitalisation, telepsychiatry could be harnessed as a tool. There are already helpline/toll free numbers in various hospitals in Nepal, which support people who have suicidal ideation. Some NGOs (the Nepal Medical Corps, Danphe Care and Patan Hospital) have initiated telepsychiatry services since the COVID-19 pandemic, to compensate for the closure of out-patient care facilities. However, the effectiveness of telepsychiatry as a means of providing consultations, particularly in the absence of supervisory bodies, has yet to be explored in the country.

## Data Availability

Data availability is not applicable to this article as no new data were created or analysed in this study.
